# Abdominal subcutaneous fat area can predict 2-year survival in patients with end-stage renal disease initiating hemodialysis

**DOI:** 10.1371/journal.pone.0304486

**Published:** 2025-04-23

**Authors:** Wonjung Choi, Hyerim Park, Hwajin Park, Yoon-kyung Chang, Dae Eun Choi

**Affiliations:** 1 Nephrology, College of Medicine, The Catholic University of Korea, Seoul, South Korea; 2 Medical Science, Medical School, Chungnam National University, Daejeon, South Korea; 3 Nephrology, Chungnam National University Hospital, Daejeon, South Korea; University of Oklahoma, UNITED STATES OF AMERICA

## Abstract

Obesity and adipose tissue are commonly regarded as detrimental factors linked to adverse outcomes, including cardiovascular and metabolic diseases. However, the obesity paradox is obesity that may provide survival benefits for chronic diseases including patients undergoing hemodialysis. Fat mass can be a surrogate marker for nutrition status in patients undergoing hemodialysis. Thus, this study evaluated subcutaneous fat and all-cause mortality in patients initiating hemodialysis. A total of 123 patients initiating hemodialysis were included in this study. MATLAB (version R2014a) was used to identify subcutaneous fat area (SFA) and visceral fat area (VFA) in computed tomography images for the analysis of body composition. The survival rate was calculated using Cox regression analysis. The Kaplan–Meier survival rates were 70.0% and 85.7% in the low and high subcutaneous fat area (SFA) groups, respectively (log rank, p = 0.021). In Cox analysis, the low SFA group showed high risk for all-cause mortality than the high SFA group (hazard ratio (HR) 3.541, 95% CI 1.358–9.235, p = 0.010). In subgroup univariate analysis, the risk for all-cause mortality was higher in patients with low SFA and diabetes than those with high SFA and diabetes (HR 3.541, 95% CI 1.358–9.235, p = 0.010). In multivariate analysis, the risk for all-cause mortality was higher in patients with low SFA and diabetes than those with high SFA and diabetes (HR 4.615, 95% CI 1.484–14.351, p = 0.008). Conclusively, low SFA increases the risk of 2-year all-cause mortality, and SFA analysis can provide information for risk evaluation for patients initiating hemodialysis.

## Introduction

Obesity is a risk factor for increased cardiovascular diseases and diabetes mellitus (DM) in the general population. However, the obesity paradox is observed in chronic diseases such as advanced chronic kidney disease, which referred to the survival benefit of obesity in patients with end-stage renal disease (ESRD) [1,2]. To explain the pathophysiology of the obesity paradox, some studies have highlighted the time discrepancies between short-term mortality factors such as protein-energy wasting (PEW)-related nutrition effect and long-term factors such as obesity-related traditional mortality risk factors [1,3]. Adipose tissue can provide hemodynamic stability during hemodialysis, fat-related lipoprotein can protect against circulating endotoxin, anti-inflammatory cytokine, and less pro-inflammatory cytokines, and subcutaneous fat can sequestrate the uremic toxins [1,4,5]. Previous studies have emphasized that body mass index (BMI) and serum creatinine and muscle mass evaluation are used as nutritional status indicators to show the survival benefits.BMI cannot distinguish between subcutaneous fat, visceral fat, and muscles [6]. This problem can be exacerbated during hemodialysis (HD), in which the muscles may account for a smaller percentage of the body weight because of edema and an overhydrated state [7]. Furthermore, in patients initiating hemodialysis, uremic symptoms such as anorexia can lead to sarcopenia and cachexia due to insufficient dietary protein intake and PEW syndrome [8]. Overhydration or PEW state in patients initiating hemodialysis makes it difficult to properly assess BMI and muscle mass, and the initial measurement of this modality cannot predict the patient’s prognosis. On the contrary, fat tissue analysis can be assessed relatively accurately than the muscles even in patients starting hemodialysis. In CT analysis, the radiation attenuation of tissues is measured in Hounsfield units (HU).

the HU range for skeletal muscle is -29 to +150 HU, overlapping with the HU value for water (0 HU). As a result, in conditions characterized by excessive fluid, such as edema, there is a potential for overestimation in the measurement of muscle attenuation [9]. In general population, subcutaneous fat have beneficial metabolic effects and possibly reflects overall nutritional status as energy storage organ [10]. McLaughlin et al. found that despite nearly identical mean BMI values, visceral adipose tissue, quantified by CT results, was significantly higher in the insulin resistance group, whereas subcutaneous adipose tissue was significantly lower in insulin resistance group [11]. Tanko et al. reported that subcutaneous fat, determined by DXA, had an independent negative correlation with both atherogenic metabolic risk factors, such as glucose and lipid metabolites, and aortic calcification, assessed by lateral radiograph findings in old age women [12]. In hemodialysis patients, Huang et al.reported that higher triceps skinfold thickness was significantly associated with decreased risk of mortality in hemodialysis patients [13]. Although direct measurement of subcutaneous fat was not performed, considering that skinfold thickness reflect subcutaneous fat, their results indicated that more higher subcutaneous fat mass may be associated with better survival in hemodialysis patients.. However, the direct association between subcutaneous fat and mortality in patients with ESRD is not established yet. Thus, this study aimed to investigate the association between subcutaneous fat and all-cause mortality in patients initiating hemodialysis.

## Methods

### Study participants

**Fig 1:**
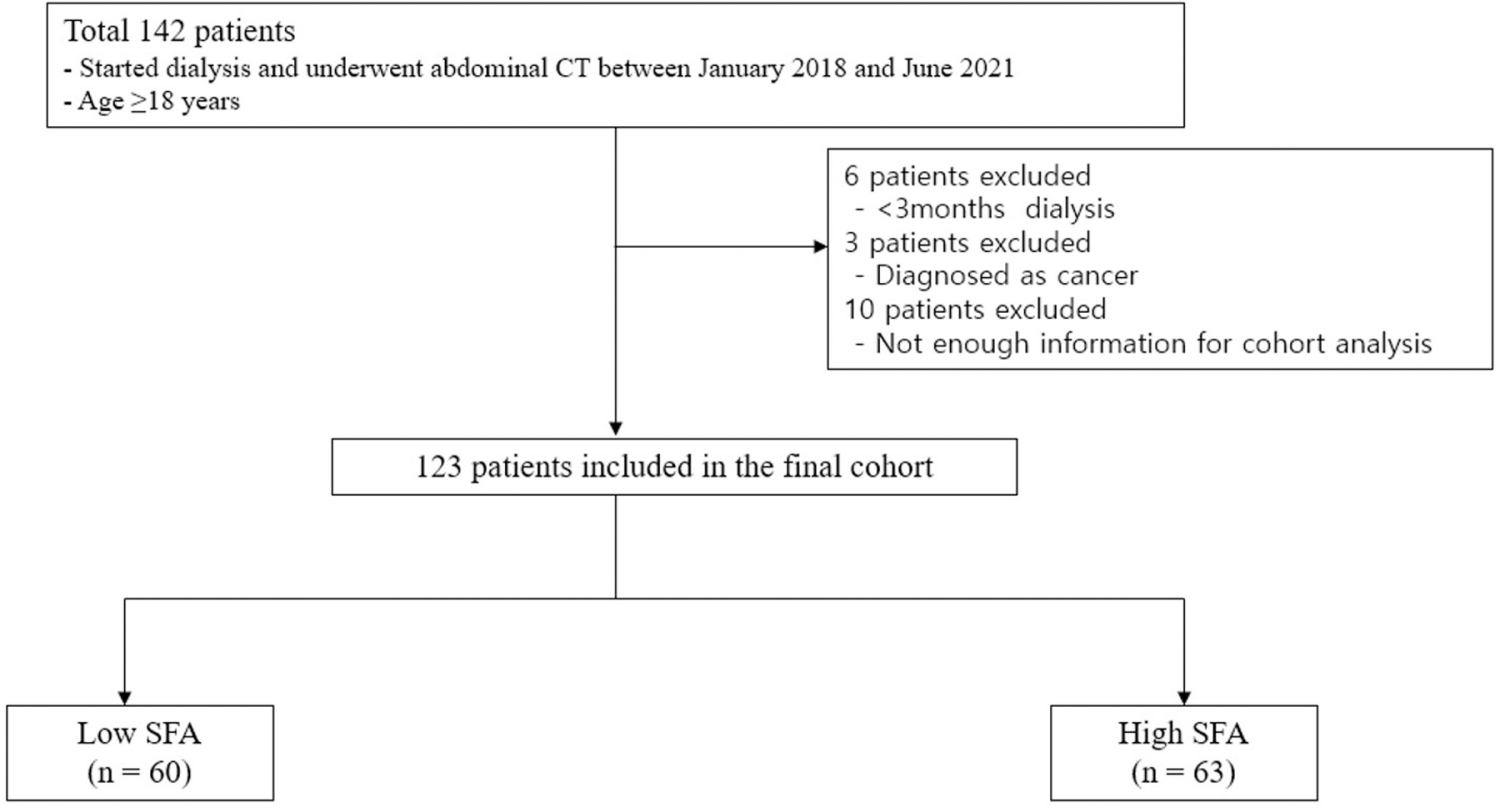
Flow diagram for study population. Abbreviation. CT, computed tomography; SFA, subcutaneous fat analysis.

A total of 123 patients who only started maintenance HD and underwent abdominal computed tomography (CT) at Daejeon St. Mary Hospital between January 2018 and June 2021 were enrolled. Patients ages ≥18 years were included. Patients diagnosed with acute kidney injuries, were undergoing hemodialysis for <3 months, and had <3 months of survival and malignancy were excluded. Fig 1 shows the flow chart for this study population. This study adhered to the principles of the Declaration of Helsinki, and the study protocol was approved by the ethics committee of Daejeon St. Mary Hospital (IRB: DC21RISI0088).

### Data collection, definitions, and measurements

**Fig 2:**
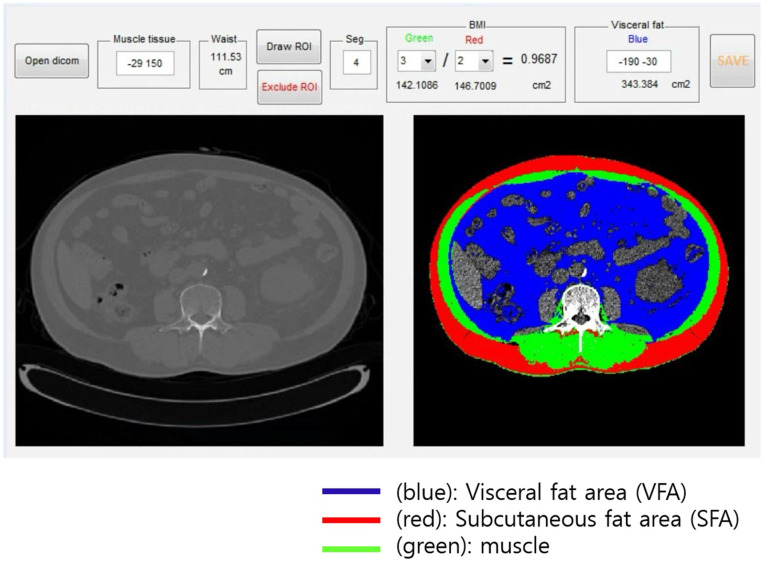
Semiquantitative body mass index and subcutaneous fat computed tomography analysis.

The following patient information was collected from the medical records: age, sex, initial HD date, diabetes mellitus (DM), hypertension (HTN), body mass index (BMI), weight, and height. Blood samples were collected from the patients before the initiation of the first HD session, and data obtained within 1 month of CT were used. Subcutaneous fat area (SFA) and visceral fat area (VFA) were determined using abdominal CT. The analysis was conducted using open-source software to identify SFA and VFA in CT images for the body composition analysis (Fig 2). MATLAB version R2014a (Mathworks Inc., Natick, MA) was used (https://sourceforge.net/projects/muscle-fat-area-measurement/) [14]. The CT image was obtained at the lumbar level 3 (L3) single cross-section, and a semi-automatic software was utilized with cut-off levels (300 to −50 HU for fat tissue; +10 to +100 HU for muscle) to extract the muscle and two adipose areas. The CT examinations were conducted using multi-detector row CT (MDCT) scanners, specifically Siemens SOMATOM Definition Flash (dual source 128) and Siemens SOMATOM Force (dual source 192). The scanning parameters were set at 90 kVp, 109–120 mAs, and a 3 mm slice thickness. To evaluate nutritional status, the controlling nutritional status (CONUT) score and the prognostic nutrition index (PNI), a simplified nutritional assessment tool using only laboratory data, were used. The CONUT score is shown in Table 1, and PNI was calculated using the following calculation method [15,16].

**Table 1:** Nutritional status by the CONUT score.

Parameters	Normal	Light	Moderate	Severe
Albumin (g/dL)	≥3.50	3.00–3.49	2.50–2.99	<2.50
Score	0	2	4	6
Total Lymphoyte/µL	≥1600	1200–1599	800–1199	<800
Score	0	1	2	3
Total cholesterol mg/dL	≥180	140–179	100–139	<100
Score	0	1	2	3
Total score	0–1	2-4	5–8	9–12

Abbreviation: CONUT, controlling nutritional status.


$$\text{PNI=10×serum albumin}\left(\text{g/dL}\right)\text{+0.005×lymphocyte count}\left(\text{/m}{\text{m}}^{\text{3}}\right)$$

In this study, BMI was calculated from the initial weight and height as follows:


$$\text{BMI=weight}\left(\text{kg}\right)\text{/heigh}{\text{t}}^{\text{2}}\left({\text{m}}^{\text{2}}\right)\text{.}$$

### Follow-up study

The study endpoint was all-cause mortality. The patients were divided into two groups according to each cutoff point. All-cause mortality was determined using the use of Kaplan–Meier (K-M) curve. Patients were followed up for 2 years.

### Statistical analysis

Continuous variables were compared using the t-test. Categorical variables are expressed as numbers with percentages and were compared using the chi-squared test. A p-value of <0.05 was considered statistically significant. These variables are presented as mean ± standard deviation for those with normal distribution and median (interquartile range [IQR]) for those with a non-normal distribution. K-M analysis was used to plot survival curves between the groups, and the log-rank method was used as determining statistical significance. The survival rate was calculated using Cox regression analysis, and univariate, and multivariate analyses were performed. All statistical analyses were performed using IBM SPSS Statistics version 23.0 (IBM Corp., NY, USA).

## Results

### Baseline characteristics

The baseline characteristics of the study patients are shown in Table 2. The mean SFA, VFA, and BMI were 108.17 ± 26.37, 143.87 ± 91.38, and 23.83 ± 4.52, respectively. SFA groups were divided into two groups by cut-off value calculated by ROC analysis (S1 Fig). The mean patient age was 64.12 ± 14.33 years. The mean age of the high SFA group was lower than the low SFA group, with 61.07 ± 14.36 and 67.31 ± 13.70, respectively. Of all patients, 69 (56.1%) were male. The proportion of male patients was higher in the high SFA group (81.0%) than in the low SFA group (30.0%).

**Table 2:** Baseline characteristics between the subcutaneous fat area (SFA) groups.

	Subcutaneous fat area (cm^2^)			
	Total (n = 123)	low SFA group (n = 60)	high SFA group (n = 63)	*P* *
Age (years)	64.12 ± 14.33	**67.31 ± 13.70**	**61.07 ± 14.36**	**0.015**
Sex (male, %)	69(56.1)	**18(30.0)**	**51(81.0)**	**<0.001**
Waist (cm)	91.08 ± 12.29	**86.63 ± 12.05**	**95.31 ±11.03**	**<0.001**
BMI (kg/m^2^)	23.83 ± 4.52	**21.52 ± 3.62**	**25.93 ±4.24**	**<0.001**
SFA (cm^2^)	108.17 ± 26.37	**86.43 ± 14.44**	**128.88 ±16.66**	**<0.001**
VFA (cm^2^)	143.87 ± 91.38	128.20 ± 85.72	158.79 ±94.73	0.063
Diabetes mellitus (yes, %)	84(68.3)	39(65.0)	45(71.4)	0.561
Hypertension (%)	102(82.9)	**45(75.0)**	**57(90.5)**	**0.003**
Triglyceride (mg/dL)	143.79 ± 91.30	133.07 ± 91.32	153.48 ±90.92	0.313
Total cholesterol (mg/dL)	143.08 ± 55.82	144.69 ± 46.59	151.09 ±63.12	0.686
HDL-cholesterol(mg/dL)	41.80 ±16.35	42.85 ± 15.14	40.85 ±17.44	0.509
WBC (10^3^/mm^3^)	8.73 ± 4.99	9.57 ± 6.35	7.91 ±3.12	0.067
Lymphocyte# (10^3^/mm^3^)	1.24 ± 0.56	**1.07 ±0.54**	**1.39 ±0.53**	**0.001**
Hemoglobin (g/dL)	9.49 ± 1.48	9.51 ± 1.57	9.47 ± 1.39	0.892
Hs-CRP (mg/dL)	1.69 ± 3.65	**2.36 ± 4.71**	**1.05 ± 2.08**	**0.046**
Total protein (g/dL)	6.21 ± 0.98	6.12 ± 1.13	6.30 ± 0.81	0.319
Albumin (g/dL)	3.58 ± 0.64	3.54 ± 0.63	3.62 ± 0.64	0.490
BUN (mg/dL)	80.70 ± 29.88	82.37 ± 33.44	79.11 ± 26.22	0.547
Creatinine (mg/dL)	7.34 ± 3.29	**6.56 ± 2.98**	**8.08 ± 3.43**	**0.010**
eGFR (mL/min/1.73 m^2^)	9.65 ± 8.15	**11.15 ± 10.77**	**8.22 ± 4.03**	**0.046**
Glucose (mg/dL)	150.87 ± 69.70	152.00 ± 78.34	149.0 ± 60.93	0.762
HbA1c (%)	7.44 ± 8.89	8.30 ± 12.75	6,64 ±1.42	0.309
Uric acid (mg/dL)	7.54 ± 6.91	6.79 ± 2.53	8.24 ± 9.32	0.247
Ferritin (ng/mL)	377.46 ± 340.34	415.62 ± 361.15	339.93 ± 317.10	0.227
Nutrition status				
CONUT score	4.24 ± 2.52	4.64 ± 2.88	3.87 ±2.09	0.092
PNI	42.08 ± 7.53	40.84 ± 8.17	43.26 ±6.71	0.076

Abbreviations: BMI, body mass index; SFA, subcutaneous fat area; VFA, visceral fat area; HDL-C, high-density lipoprotein cholesterol; WBC, white blood cells; hs-CRP, high-sensitivity C-reactive protein; BUN, blood urea nitrogen; eGFR, estimated glomerular filtration rate; CONUT, controlling nutritional status; PNI, prognostic nutrition index

*p value is compared between low SFA group and high SFA group.

### Abdominal fat and mortality

**Fig 3:**
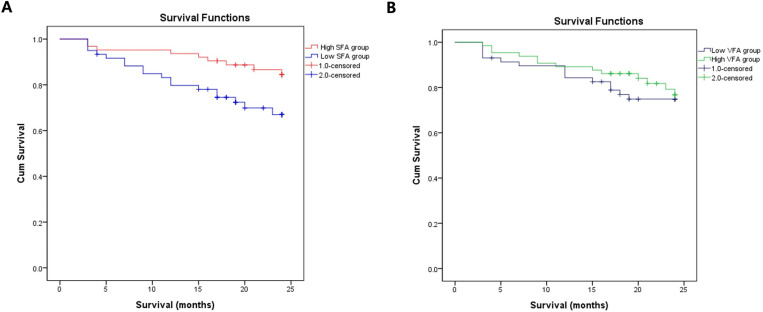
Kaplan–Meier survival curve of SFA groups and VFA groups. (A) low and high SFA groups, (B)low and high VFA groups.

During the 2-year follow-up period, 27 patients died. The K-M survival rates were 70% and 85.7% in the low and high SFA groups, respectively (log rank, p = 0.021), respectively. However, the K-M survival rates did not show the significant differences between the high VFA and low VFA groups (Fig 3). In the univariate Cox proportional hazards analysis, the low SFA group was significantly at high risk for all-cause mortality (hazard ratio [HR] 3.105, 95% confidence interval [CI] 1.356–7.113, p = 0.005). After adjusting for age, sex, DM, HTN, hemoglobin, blood urea nitrogen(BUN), creatinine (Cr), estimated glomerular filtration rate, albumin, glucose, total cholesterol(Tchol), triglycerides(TG), C-reactive protein(CRP), VFA, CONUT score, and PNI, the statistical significance of low SFA influencing mortality was lost (Table 3). The Spearman correlation analysis conducted to examine the association of SFA with PNI (r = 0.106, p = 0.244) and the CONUT score (r = −0.031, p = 0.738) indicated that higher SFA was positively correlated with the nutrition status, though not statistically significant (S2 Fig).It was evaluated whether the Tchol, TG, and low-density lipoprotein (LDL) were related to SFA and these lipid profiles were related to survival. Tchol, TG, and LDL showed a positive correlation with SFA; however, there was no statistical significance (S3 Fig). Additionally, Tchol, TG, and LDL were not correlated with patient’s survival (S4 Fig).

**Table 3:** Crude and multivariate Cox regression analyses.

	Hazard ratio (95% CI)	*p* value
Crude	**3.105 (1.356–7.113)**	**0.005**
Model 1	1.668(0.817-3.406)	0.160
Model 2	1.585(0.770-3.264)	0.212

Model 1 includes age, sex, SFA grade, VFA, DM, HTN, Hb, BUN, Cr, total protein, albumin, glucose, Tchol, TG, HDL-CCRP, and BMI.

Model 2 includes all variables in Model 1 + CONUT score and PNI.

Abbreviations: SFA, subcutaneous fat area; VFA, visceral fat area; DM, diabetes mellitus; HTN, hypertension; Hb, hemoglobin; BUN, blood urea nitrogen; Cr, creatinine;Tchol, total cholesterol;TG, triglyceride; HDL-C, high-density lipoprotein cholesterol; CRP, C-reactive protein; CONUT, controlling nutritional status; PNI, prognostic nutrition index

*p value is compared between low SFA group and high SFA group.

### Subgroup analysis of the HR for all-cause mortality

We determined whether each SFA group had a greater effect on mortality according to age, BMI, DM status, and nutritional status. In the subgroup univariate analysis, the low SFA group with diabetes was at a higher risk for all-cause mortality (HR 3.541, 95% CI 1.358–9.235, p = 0.010) than the high SFA group with diabetes.). In subgroup multivariate analysis, patients with low SFA and DM were at higher risk for all-cause mortality than those with high SFA and DM (HR 4.615, 95% CI 1.484–14.351, p = 0.008) (Table 4). On the contrary, age, BMI, PNI and CONUT subgroup analysis were not significant between high and low SFA groups (Table 4 and S5 Fig).

**Table 4:** Association between the SFA groups and subgroup analysis.

		Univariate hazard ratio (95% CI)	*p* value	Multivariate hazard ratio (95% CI)	*p* value
Age <65	High SFA	ref.		ref.	
	Low SFA	1.948(0.436–8.706)	0.383	–	–
Age ≥65	High SFA	ref.		ref.	
	Low SFA	2.289(0.875–5.988)	0.091	2.171(0.629–7.494)	0.220
BMI <25	High SFA	ref.		ref.	
	Low SFA	3.089(0.870–10.97)	0.081	–	–
BMI ≥25	High SFA	ref.		ref.	
	Low SFA	3.744(0.931–15.051)	0.063	–	–
PNI <41	High SFA	ref.		ref.	
	Low SFA	1.983(0.707–5.564)	0.193	3.041(0.320–28.916)	0.333
PNI ≥41	High SFA	ref.		ref.	
	Low SFA	2.136(0.569–8.017)	0.261	–	–
CONUT <5	High SFA	ref.		ref.	
	Low SFA	2.762(0.805–9.482)	0.106	–	–
CONUT ≥5	High SFA	ref.		ref.	
	Low SFA	2.469(0.785–7.768)	0.122	–	–
Non-DM	High SFA	ref.		ref.	
	Low SFA	2.301(0.442-11.978)	0.322	0.000	0.868
DM	High SFA	ref.		ref.	
	Low SFA	**3.541(1.358-9.235)**	**0.010**	**4.615(1.484-14.351)**	**0.008**

Multivariate: age, sex, SFA grade, DM, HTN, Hb, BUN, Creatinine, total protein, albumin, glucose, Tchol, TG, HDL-C, CRP, BMI, CONUT, PNI

## Discussion

In this study, we showed that the survival rate of patients initiating hemodialysis with higher SFA was 3.105 times higher than those with lower SFA.

Adipose tissues act as storage of energy fuel; during hemodialysis, which is highly energy demanding, fat can be an energy source [5]. Fat is divided into subcutaneous fat and visceral fat, and according to other studies, subcutaneous fat has an advantage over visceral fat [17,18]. Compared with subcutaneous adipose tissue, visceral fat produces higher levels of inflammatory cytokines [19]. Subcutaneous tissue secretes adiponectin related to fewer anti-inflammatory cytokines, such as tumor necrosis factor (TNF)-alpha receptors, and adiponectin attenuates TNF-alpha, and IL-6 production, suppresses NF-kB activation, and regulates inflammation response [1,5,20].

Higher SFA in patients on hemodialysis has indirectly suggested better survival [13]. Consistent with these studies, our study showed that high SFA showed better survival in patients with hemodialysis. In the early stages of hemodialysis, the patient’s actual lean body weight and current weight can differ due to edema or overhydration. Moreover, BMI does not discriminate between body fat mass and lean mass and poorly reflects body fat distribution [21]. Thus, BMI may not be a favorable measurement tool in early stages of hemodialysis. Furthermore, patients with nephrotic-range proteinuria and advanced chronic kidney disease are associated with a hypercatabolic status and exacerbated PEW and loss of muscle mass [22]. PEW can induce malnutrition and worsen nutritional status, leading to underestimation of nutritional assessment. Aditionally, overhydration induces impaired tissue oxygen delivery impairment due to interstitial edema and increases hypoxic damage, inflammatory mediators, and oxidative stress [23]. Overhydration hinders accurate measurement of nutritional and body composition status [24]. Conversely, high fat mass has been suggested to have favorable impact on fluid control in patients on hemodialysis [25]. Thus, the fat tissue analysis can be assessed with relatively better accuracy than muscles even in patients initiating hemodialysis. Therefore, SFA measurement should be considered as a survival-related factor to be determined in patients initiating HD.

It has been known that diabetic patients undergoing hemodialysis show higher mortality than do nondiabetic patients undergoing hemodialysis [26]. In our study, high SFA in diabetic patients undergoing hemodialysis was suggested as a factor associated with survival. In general population, studies have shown that subcutaneous fat is beneficial in patients with DM [17,27]. And subcutaneous fat has been associated with reduced risk in female patients with DM [10]. However, as a theoretical background for estimating that subcutaneous fat as an important survival factor in patients initiating hemodialysis, controlling nutritional and hydration status is challenging for patients with DM and subcutaneous fat may act as a nutritional mediator to protect from inflammation and help to control hydration status.

Although not statistically significant in this study, it tended to play an important role in the survival of SFA in patients aged ≥65 years and in those with high BMI (≥25). These results suggest that a higher presence of SFA may be beneficial in patients who are older and those who are obese; however, future studies in larger cohorts are warranted. Tchol, TG, and LDL are traditionally known as risk factors for cardiovascular disease survival [28–30]. However, in this study, Tchol, TG, and LDL factors were not influencing survival in patients initiating hemodialysis, rather they showed a positive correlation with SFA. Low levels of Tchol, TG, and LDL can indirectly suggest that patients’ survival is not good. This further indicates that similar to patients on maintenance hemodialysis, in the patients initiating hemodialysis, T chol, TG, and LDL levels are considered important factors for representing the nutritional status than affecting endothelial cells of blood vessels. SFA and nutritional status (CONUT, PNI) were not statistically significant, but showed a tendency of positive correlation.

Overall, it was found that among the factors representing nutritional status and fat mass in patients initiating hemodialysis, SFA plays an important role in the patients’ survival.

This study has several limitations. First, it is a retrospective study with a relatively small sample size and conducted on a single-center setting. It has the limitation of not including a diverse range of ethnicities. Second, the hemodialysis modality was not considered. Third, the cause of death was not considered, and all-cause mortality was analyzed in this study. Fourth, the followed laboratory data did not consider as the changes in nutritional status during follow-up, which may affect the prognosis. In this study, abdominal fat analysis was based on abdominal CT, which may not reflect adipose tissue in areas other than the abdomen. Additionally, a head-to-head comparison of measurement was not conducted through CT with dual X-ray energy, which is a known assessment tool for body composition status.

Finally, this retrospective cohort study cannot elucidate the pathogenesis of the results and require further evaluation.

## Conclusions

A low SFA increases the risk of 2-year all-cause mortality in patients initiating hemodialysis, especially in patients with DM suggesting that early SFA analysis may be utilized to predict mortality in these populations.

## Supporting information

S1 Fig ROC curve of VFA and SFA. (DOCX)

S2 Fig Spearman correlation between subcutaneous fat and nutrition status. (A) correlation between SFA and PNI, (B) correlation between SFA and CONUT.
(DOCX)

S3 Fig Spearman correlation between SFA and lipid profiles. (A) Total cholesterol, (B) Triglyceride (TG), (C) LDL-cholesterol.
(DOCX)

S4 Fig ROC curve and Kaplan Meier curve for Lipid profiles. (A) ROC curve of Lipid profiles, (B) Kaplan Meier curve of total cholesterol (total chol), (C) Kaplan Meier curve of triglyceride(TG), (D) Kaplan Meier curve of LDL cholesterol.
(DOCX)

S5 Fig Forest plot of hazard ratio for all-cause mortality by prespecified subgroups. (DOCX)

S1 File Raw laboratory data of the patient. (XLSX)

S1 Checklist STROBE Statement—checklist of items that should be included in reports of observational studies. (DOCX)
