# Supplementary material for: Abdominal subcutaneous fat area can predict 2-year survival in patients with end-stage renal disease initiating hemodialysis
Source: PLoS One. 2025 Apr 23;20(4):e0304486. doi: 10.1371/journal.pone.0304486 (PMC12017507; doi:10.1371/journal.pone.0304486)
Supplement: S1 Fig — (DOCX) [file pone.0304486.s001.docx]

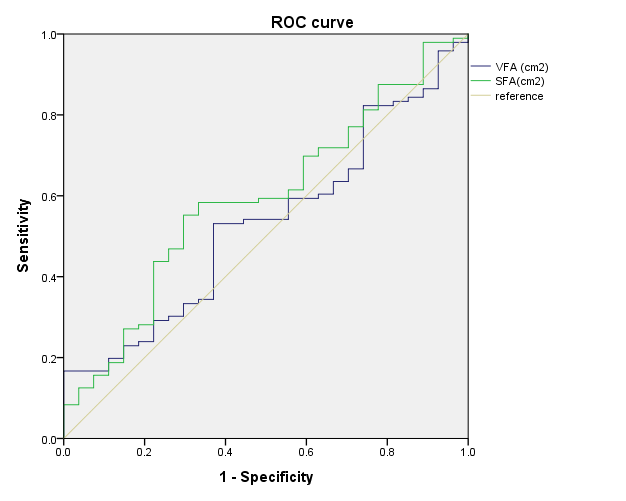


**S1 Fig.** ROC curve of VFA and SFA. Abbreviation: ROC, receiver operating characteristic; VFA, visceral fat area; SFA, subcutaneous fat area.
