# Supplementary material for: Abdominal subcutaneous fat area can predict 2-year survival in patients with end-stage renal disease initiating hemodialysis
Source: PLoS One. 2025 Apr 23;20(4):e0304486. doi: 10.1371/journal.pone.0304486 (PMC12017507; doi:10.1371/journal.pone.0304486)
Supplement: S2 Fig — (A) correlation between SFA and PNI, (B) correlation between SFA and CONUT. (DOCX) [file pone.0304486.s002.docx]

**A**

**
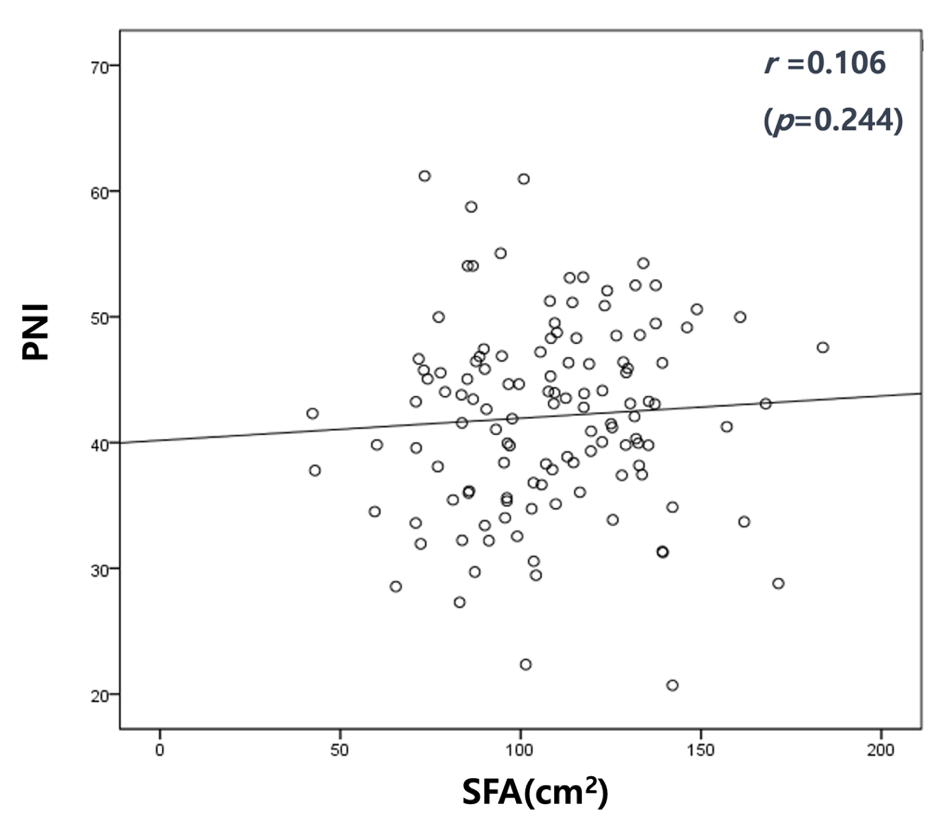
**

**B**

**
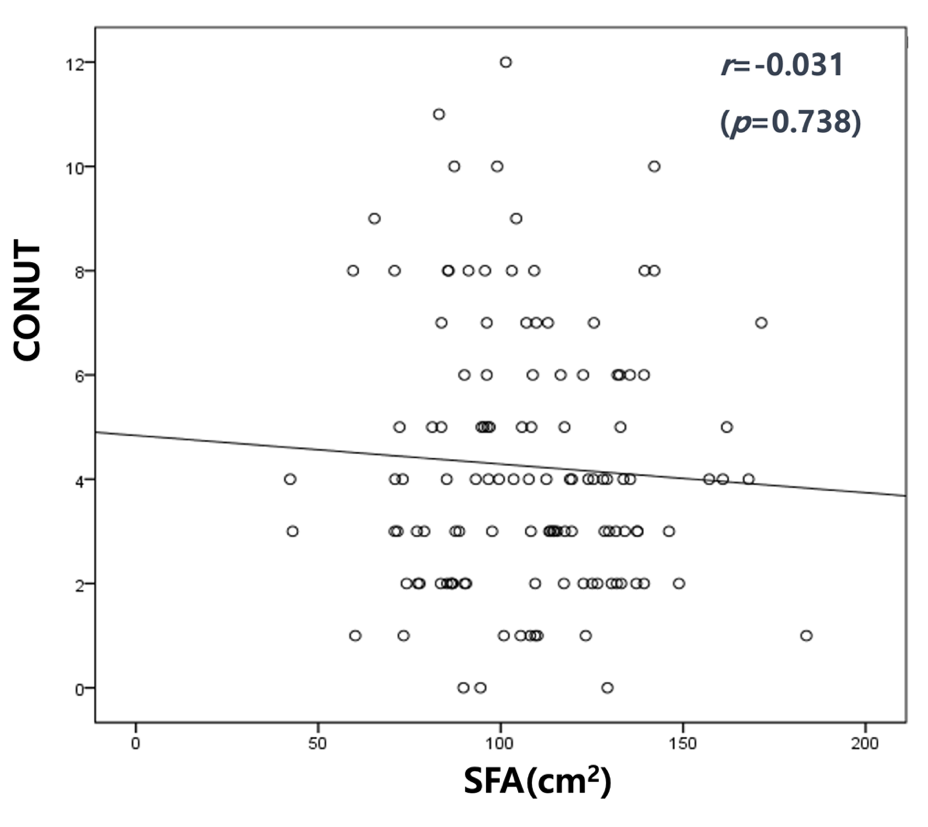
**

**S2 Fig.** Spearman correlation between subcutaneous fat and nutrition status. (A) correlation between SFA and PNI, (B) correlation between SFA and CONUT. Abbreviation: SFA, subcutaneous fat area; PNI, prognostic nutrition index; CONUT, controlling nutritional status.
