# Supplementary material for: Abdominal subcutaneous fat area can predict 2-year survival in patients with end-stage renal disease initiating hemodialysis
Source: PLoS One. 2025 Apr 23;20(4):e0304486. doi: 10.1371/journal.pone.0304486 (PMC12017507; doi:10.1371/journal.pone.0304486)
Supplement: S3 Fig — (A) Total cholesterol, (B) Triglyceride (TG), (C) LDL-cholesterol. (DOCX) [file pone.0304486.s003.docx]

**A**


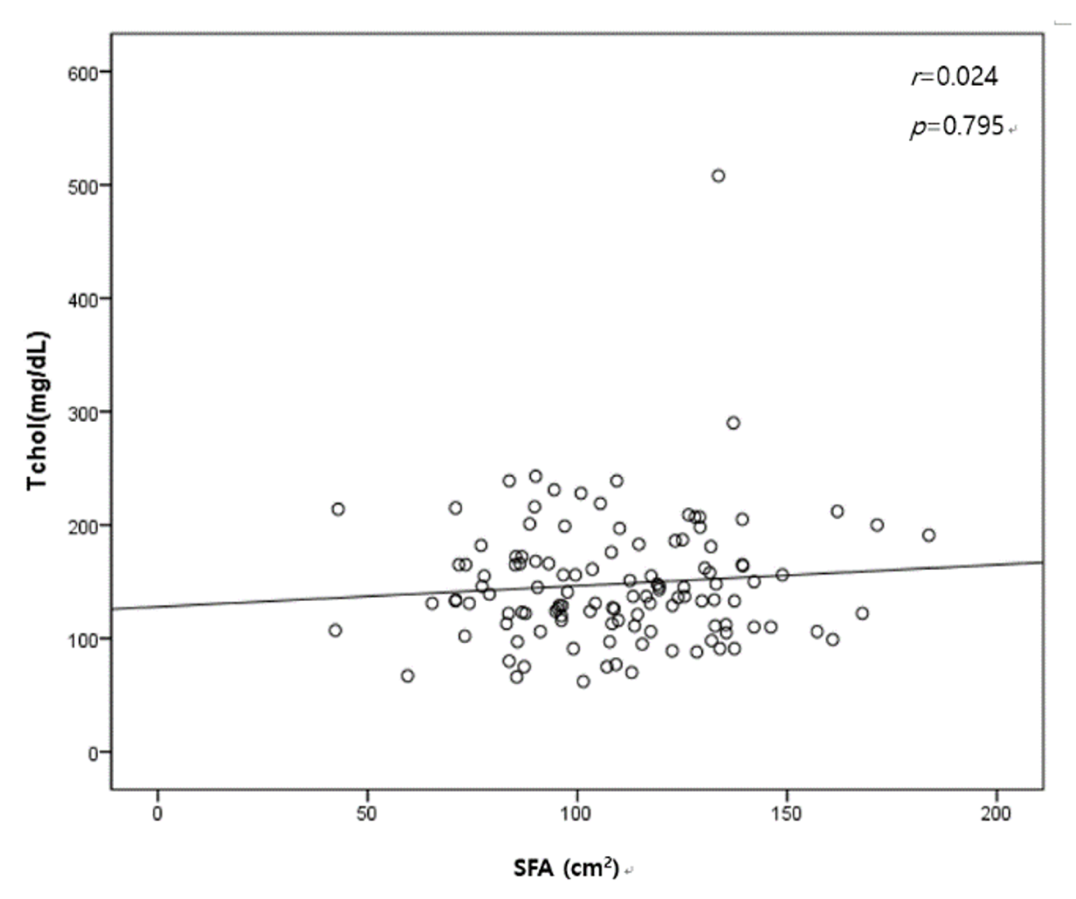


**B**


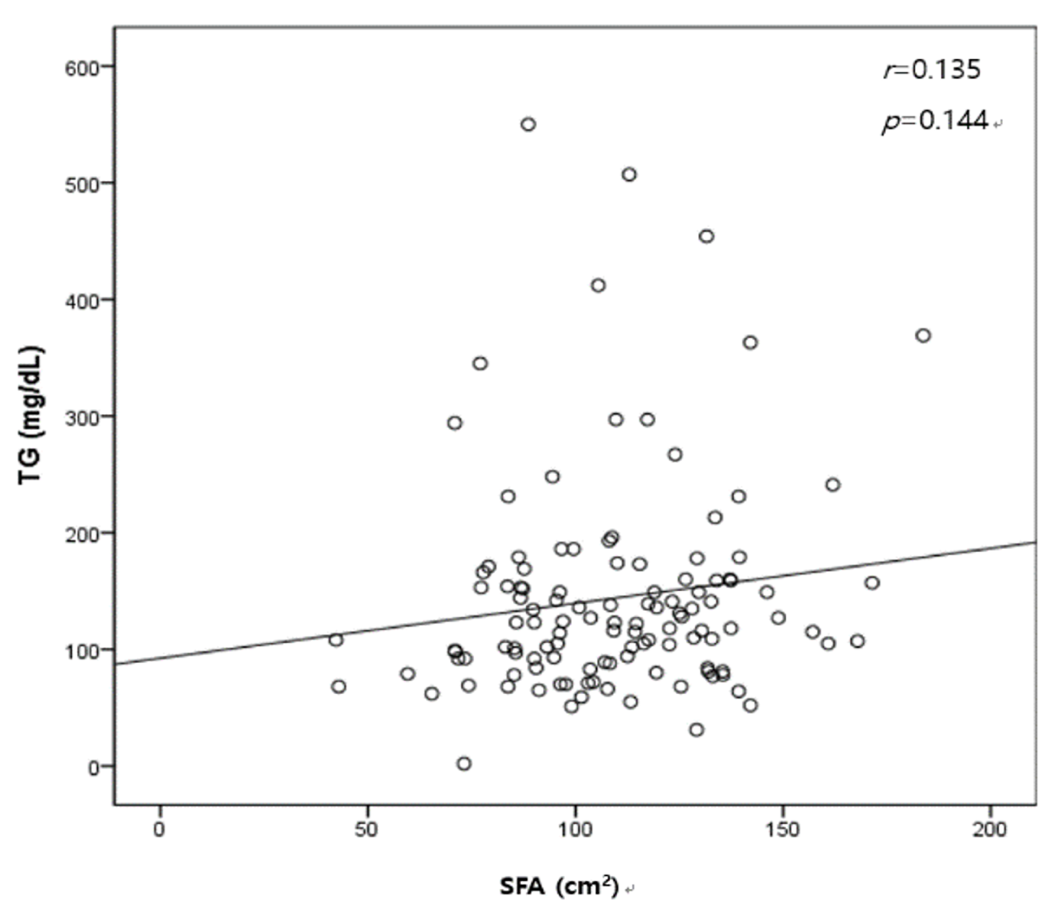


**C**

**
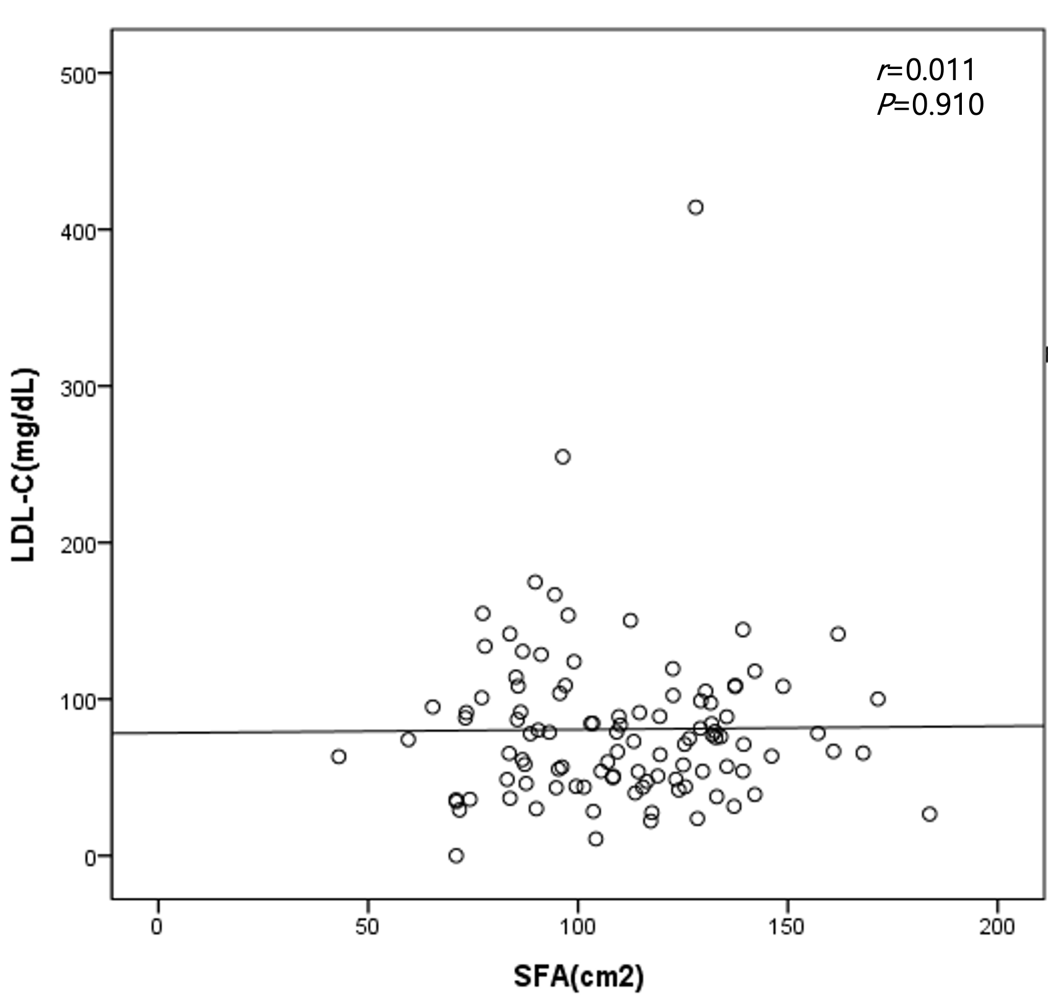
**

**S3 Fig.** Spearman correlation between SFA and lipid profiles. (A) Total cholesterol, (B) Triglyceride (TG), (C) LDL-cholesterol.
