# Supplementary material for: Abdominal subcutaneous fat area can predict 2-year survival in patients with end-stage renal disease initiating hemodialysis
Source: PLoS One. 2025 Apr 23;20(4):e0304486. doi: 10.1371/journal.pone.0304486 (PMC12017507; doi:10.1371/journal.pone.0304486)
Supplement: S4 Fig — (A) ROC curve of Lipid profiles, (B) Kaplan Meier curve of total cholesterol (total chol), (C) Kaplan Meier curve of triglyceride(TG), (D) Kaplan Meier curve of LDL cholesterol. (DOCX) [file pone.0304486.s004.docx]

**A**


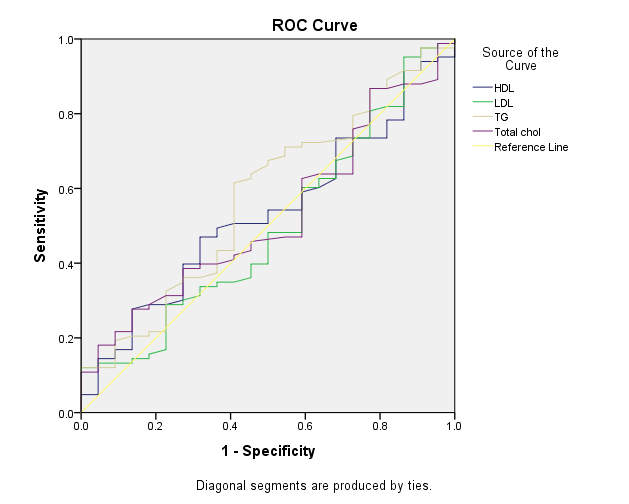


**B**


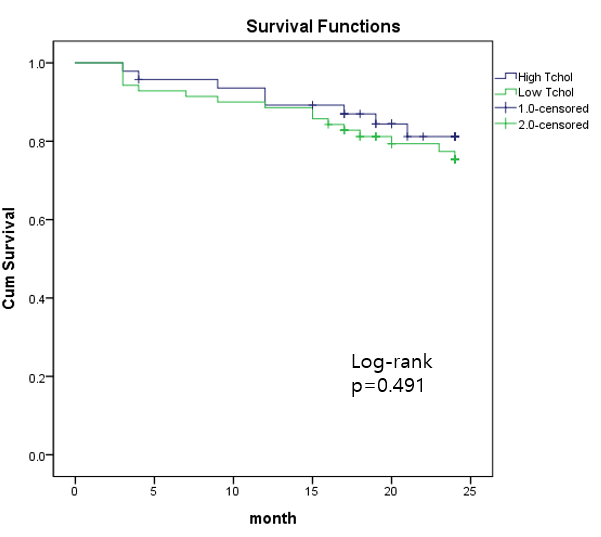


**C**

**
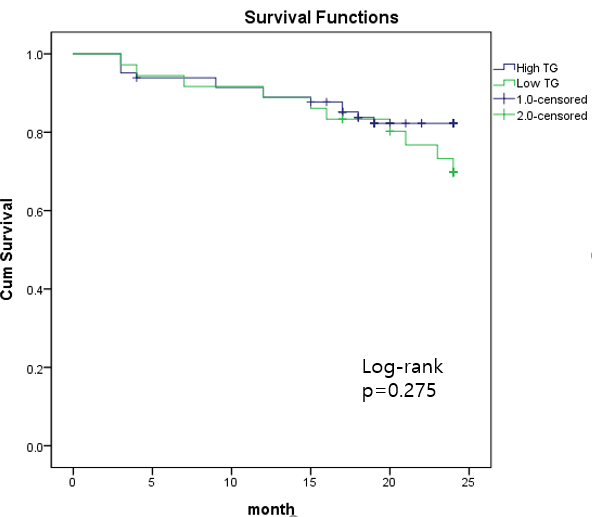
**

**D**


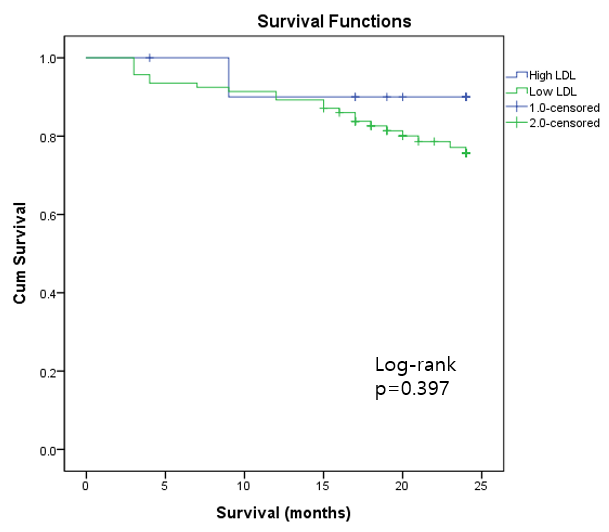


**S4 Fig.** ROC curve and Kaplan Meier curve for Lipid profiles. (A) ROC curve of Lipid profiles, (B) Kaplan Meier curve of total cholesterol (total chol), (C) Kaplan Meier curve of triglyceride(TG), (D) Kaplan Meier curve of LDL cholesterol
