# Supplementary material for: Abdominal subcutaneous fat area can predict 2-year survival in patients with end-stage renal disease initiating hemodialysis
Source: PLoS One. 2025 Apr 23;20(4):e0304486. doi: 10.1371/journal.pone.0304486 (PMC12017507; doi:10.1371/journal.pone.0304486)
Supplement: S5 Fig — (DOCX) [file pone.0304486.s005.docx]

**S5 Fig.** Forest plot of hazard ratio for all-cause mortality by prespecified subgroups.
